# *Sansevieria roxburghiana* Schult. & Schult. F. (Family: Asparagaceae) Attenuates Type 2 Diabetes and Its Associated Cardiomyopathy

**DOI:** 10.1371/journal.pone.0167131

**Published:** 2016-11-28

**Authors:** Niloy Bhattacharjee, Ritu Khanra, Tarun K. Dua, Susmita Das, Bratati De, M. Zia-Ul-Haq, Vincenzo De Feo, Saikat Dewanjee

**Affiliations:** 1 Advanced Pharmacognosy Research Laboratory, Department of Pharmaceutical Technology, Jadavpur University, Kolkata, India; 2 Phytochemistry and Pharmacognosy Research Laboratory, Department of Botany, University of Calcutta, Kolkata, India; 3 Office of Research, Innovation and Commercialization, Lahore College for Women University, Lahore, Pakistan; 4 Department of Pharmacy, University of Salerno, Fisciano, Salerno, Italy; Stellenbosch University, SOUTH AFRICA

## Abstract

**Background:**

*Sansevieria roxburghiana* Schult. *&* Schult. F. (Family: Asparagaceae) rhizome has been claimed to possess antidiabetic activity in the ethno-medicinal literature in India. Therefore, present experiments were carried out to explore the protective role of edible (aqueous) extract of *S*. *roxburghiana* rhizome (SR) against experimentally induced type 2 diabetes mellitus (T2DM) and its associated cardiomyopathy in Wistar rats.

**Methods:**

SR was chemically characterized by GC-MS analysis. Antidiabetic activity of SR (50 and 100 mg/kg, orally) was measured in high fat diets (ad libitum) + low-single dose of streptozotocin (35 mg/kg, intraperitoneal) induced type 2 diabetic (T2D) rat. Fasting blood glucose level was measured at specific intermissions. Serum biochemical and inflammatory markers were estimated after sacrificing the animals. Besides, myocardial redox status, expressions of signal proteins (NF-κB and PKCs), histological and ultrastructural studies of heart were performed in the controls and SR treated T2D rats.

**Results:**

Phytochemical screening of the crude extract revealed the presence of phenolic compounds, sugar alcohols, sterols, amino acids, saturated fatty acids within SR. T2D rats exhibited significantly (p < 0.01) higher fasting blood glucose level with respect to control. Alteration in serum lipid profile (p < 0.01) and increased levels of lactate dehydrogenase (p < 0.01) and creatine kinase (p < 0.01) in the sera revealed the occurrence of hyperlipidemia and cell destruction in T2D rats. T2DM caused significant (p < 0.05–0.01) alteration in the biochemical markers in the sera. T2DM altered the redox status (p < 0.05–0.01), decreased (p < 0.01) the intracellular NAD and ATP concentrations in the myocardial tissues of experimental rats. While investigating the molecular mechanism, activation PKC isoforms was observed in the selected tissues. T2D rats also exhibited an up-regulation in nuclear NF-κB (p65) in the cardiac tissues. So, oral administration of SR (50 and 500 mg/kg) could reduce hyperglycemia, hyperlipidemia, membrane disintegration, oxidative stress, vascular inflammation and prevented the activation of oxidative stress induced signaling cascades leading to cell death. Histological and ultra-structural studies of cardiac tissues supported the protective characteristics of SR.

**Conclusions:**

From the present findings it can be concluded that, SR could offer protection against T2DM and its associated cardio-toxicity via multiple mechanisms viz. hypoglycemic, antioxidant and anti-inflammatory actions.

## Introduction

Diabetes mellitus (DM), a chronic metabolic syndrome, contributes considerably in the global health crisis [1]. Amongst various types, type 2 diabetes mellitus (T2DM) constitutes > 90% of total diagnosed DM [2]. DM is characterized by persistent hyperglycemia which damages many organs and tissues via different mechanisms [3]. Amongst various anticipated mechanisms, hyperglycemia mediated oxidative stress and inductions of vascular inflammation have been found to play the key roles in diabetic pathophysiology [3,4]. Persistent hyperglycemia causes glucose auto-oxidation leading to the over-production of intercellular reactive oxidative species (ROS) viz. superoxide radical, hydrogen peroxide and hydroxide radical. The excess of ROS provides oxidative stress to the cardiomyocytes and induces cellular damage. Increased amount of ROS activates protein kinase C (PKC) and nuclear factor-κB (NF-κB). The activation of aforementioned signal molecules play key role in hyperglycemia mediated myocardial injury [3,5]. Activation of Poly ADP ribose polymerase (PARP) during diabetic state induces a down regulation of cellular NAD and ATP, leading to energy failure and cell necrosis [5]. Besides, NF-κB activation stimulates inflammatory mediators viz. interleukins (ILs), tumor necrosis factor α (TNF α), monocyte chemo-attractant protein 1 (MCP 1), intercellular adhesion molecule 1 (ICAM 1),vascular endothelial growth factor (VEGF) and thereby induces myocardial inflammation [6,7]. In spite of modern therapeutic strategies and educational programs, the incidence of T2DM is still unabated [8]. Commercially available oral hypoglycemic agents also exhibit plenty of adverse effects including congestive heart failure with glitazones [9], gastrointestinal disturbances with glucosidase inhibitors, sulfonylureas and meglitinides [10,11]. Cardiac problems and weight gain are common adverse effects of sulfonylureas [12]. Therefore, it is a vital need to develop a unique therapeutic agent for T2DM with less toxic/adverse effects. Considering several mechanisms of diabetic pathophysiology, it has been predicted that a multi-target therapeutic agent would be advantageous in the management of T2DM and its associated pathogenesis. Multi-component plant extract would offer the multimodal therapeutic values. Therefore, current study has been designed to explore the antidiabetic potential of a chemically standardize plant extract considering ethnomedicinal knowledge as reference.

*Sansevieria roxburghiana* Schult. &Schult. F. (Family: Asparagaceae), commonly known as Indian bowstring heamp, is a perennial herb with short fleshy stem and plump rootstock. This plant is distributed throughout the coastal India and other tropical and subtropical countries [13]. The roots and rhizomes of *S*. *roxburghiana* are used in the traditional medicine as the remedies for diabetes, inflammation, pains, fever, asthma, wound, hypertension, oxidative stress and rheumatism [14–19]. Since *S*. *roxburghiana* is believed to exhibit anti-inflammatory as well as antidiabetic potential, the present study has been undertaken to establish the curative efficacy of *S*. *roxburghiana* rhizomes against T2DM and its related pathogenesis in the cardiac tissues of experimental Wistar rats.

## Material and methods

### Chemicals

Streptozotocin was procured from Hi-media (Mumbai, India). Ammonium sulphate, 1-chloro-2,4-dinitrobenzene, ethylenediaminetetraacetic acid, 2,4-dinitro-phenyl-hydrazine, 5,5-di-thio-bi(2-nitrobenzoic acid), potassium dihydrogen phosphate, N-ethylmaleimide, reduced nicotinamide adenine dinucleotide, nitro blue tetrazolium, sodium pyrophosphate, phenazinemethosulphate, thiobarbituric acid, reduced glutathione, sodium azide, trichloro acetic acid and 5-thio-2-nitrobenzoic acid were obtained from Sisco Research Laboratory (India). Bradford reagent, antibodies and bovine serum albumin were procured from Sigma-Aldrich (St. Louis, USA). The kits for different assays for different biochemical parameters were purchased from Span diagnostic Ltd., India. All other reagents and chemicals used were of analytical grade.

### Preparation of extract

*S*. *roxburghiana* rhizomes were collected from the personal garden of Mr. Niloy Bhattacharjee located at Kharagpur (22.33° N, 87.32° E), India during the month of December, 2013. It is a commercially available ornamental plant in India and it is not an endangered species. The plant has been authenticated (Ref. no. CNH/Tech.II/2015/37/316 dated 20.08.2015) by the Taxonomists of Botanical Survey of India (Howrah, India). The rhizomes were dried in an incubator (40 ± 5°C, 72 h) and crushed into powder. The powdered rhizomes were extracted with water (double distilled) containing 1% of chloroform for 48 h at 30 ± 5°C with constant stirring. Particulate matters were removed by filtration and resulting extract was freeze-dried to get the powdered crude extract of *S*. *roxburghiana* rhizomes (SR, ~10.5% w/w). Lyophilized powder was dissolved in distilled water containing tween 80 (1%) before in vivo experiment.

### Phytochemical analysis

Crude extract and adonitol (internal standard) were dissolved in 50 μl methanol:water (1:1) and evaporated to dryness. GC-MS analysis was done in gas chromatography system (Agilent 5975C, USA) following the protocol detailed by Das et al [20] using HP-5MS capillary column (length 30 m plus Duraguard 10 m, film 0.25 μm, diameter 0.25 mm narrow bore). Samples (1 μl) were inserted via the split mode (ratio 1:5) onto the GC column. Metabolites were identified by comparing the fragmentation patterns of the mass spectra and retention times (Rt) with those present in Agilent Fiehn Metabolomics library using Agilent retention time locking method [20]. Automated mass spectral de-convolution and identification system was used to de-convolute GC-MS results and to categorize chromatographic peaks.

### Animals

Wistar rats (♂, 140 ± 20 g) were housed in standard polypropylene cages under standard laboratory conditions of light:dark cycle (12 h:12 h), relative humidity (55 ± 5%), temperature (25 ± 2°C), standard diet and water ad libitum. The animal experiments were performed at the Department of Pharmaceutical Technology, Jadavpur University, India (Committee for the Purpose of Control and Supervision on Experiments on Animals Reg. No. 0367/01/C/CPCSEA, University Grants Commission, Government of India, New Delhi). The animal experiment has been approved by the Jadavpur University animal ethical committee (Ref no. AEC/PHARM/1502/05/2015 dated 30.07.2015) and the principles of laboratory animals care were observed during experiment [21].

### Oral glucose tolerance test (OGTT)

Pre-acclimatized Wistar rats (overnight fasted) were divided into 3 groups (n = 6). The animals were given glucose (1.5 g/kg body weight, orally by oral gavage) [3]. Immediately after the feeding of glucose solution, 2 groups of rats were treated with SR (50 & 100 mg/kg body weight, orally by oral gavage) and 1 group of animals (normal control) were treated with 1% tween 80 (2 ml/kg, orally by oral gavage). Blood glucose levels were measured @ 0, 30, 60, and 120 min with single touch glucometer (Ascensia Entrust, Bayer Health Care, USA). Total glycemic responses have been calculated from respective areas under the curve (AUC) throughout the observation period of 120 min.

### Experimental design

T2DM was induced by high fat diets (25% protein, 17% carbohydrate and 58% fat, as %-age of total kcal) ad libitum and low-dose of streptozotocin as described by Reed et al. [22]. Briefly, the rats were fed high fat diets ad libitum for 2 weeks and then treated with single dose of streptozotocin (35 mg/kg body weight, intraperitonially) [22]. The composition (Table 1) of high fat diet has been described by Srinivasan et al. [23]. One week after streptozotocin injection, the fasting blood glucose levels were appraised and the animals exhibiting fasting blood glucose levels of 170 ± 30 mg/dl were considered to be type 2 diabetic (T2D) rats and included for the further experiments. The rats were continued with high fat diets throughout the course of the study.

**Table 1:** The composition of high fat diet [22–24].

Ingredients	Diets (g/kg body weight)
Powdered NPD	365
Lard	310
Casein	250
Cholesterol	10
Vitamin and mineral mix	60
Yeast powder	01
Sodium chloride	01

The Wister rats were divided into following groups (n = 6) and received the treatment as follows for 28 days:

Group I: Normal control rats were administered 1% tween 80 (2 ml/kg body weight, orally by oral gavage) in distilled water daily;

Group II: T2DM control rats were administered high fat diets + 1% Tween 80 (2 ml/kg body weight, orally by oral gavage) in distilled water daily;

Group III: T2D rats were administered high fat diets + SR (50 mg/kg body weight, orally by oral gavage) daily;

Group IV: T2D rats were administered high fat diets + SR (100 mg/kg body weight, orally by oral gavage) daily;

Group V: T2D rats were administered high fat diets + glibenclamide (1 mg/kg body weight, orally by oral gavage) daily [25].

A group (Group VI) has been included, in which T2D rats were administered high fat diets throughout the course of study. This group of animals served as obese control.

**Fig 1:**
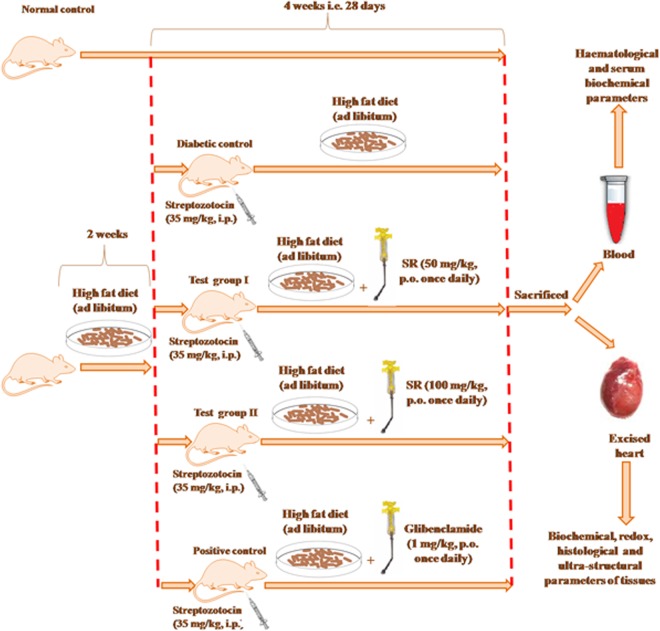
A schematic impression of experiment.

The selection of doses was entirely based on the OGTT observation. The grouping of animals was done as per the instruction given by the institutional animal ethical committee and on the basis of statistical analysis. The overall experimental design has been depicted in Fig 1. The animals were monitored at 8-hours interval for checking any sign of distress and abnormality.

### Determination of fasting blood glucose level and other serum biochemical parameters

Fasting blood glucose levels of overnight fasted rats were estimated on day 0, 1, 3, 7, 14, 21, 28 with single touch glucometer (Ascensia Entrust, Bayer Health Care, USA). After 28 days of treatment, animals were exposed to CO_2_ euthanasia and sacrificed by cervical dislocation [26]. Before sacrificing, the blood samples were obtained from retro-orbital venous plexus for serum biochemical assays. Retro-orbital bleeding was carried out without general anesthesia, however, tetracaine (0.5%) ophthalmic anesthetic drop was applied prior to the blood collection. The lactate dehydrogenase (LDH), creatine kinase (CK), HDL cholesterol, triglycerides and total cholesterol contents were measured by commercial kits (Span Diagnostic Limited, India) following the protocol mentioned by the manufacturer. LDL cholesterol was estimated by using Friedewald’s equation (LDL cholesterol = Total cholesterol–Triglycerides/5 –HDL cholesterol) [27]. Triglyceride/5 is considered to be the equivalent to VLDL cholesterol level [28]. Troponin I and T contents were determined by ELISA kits (Kamiya Biomedical Company, USA). IL 1β, IL 6 and TNF α contents were analyzed by ELISA kits (Fisher Thermo Scientific Co., USA). Nayak and Pattabiraman’s [29] method was followed to assess the glycosylated hemoglobin concentration. Insulin concentration was measured by ELISA kits (Sigma-Aldrich, USA). Homeostatic model assessments viz. HOMA-IR and HOMA-β scores were calculated employing to the following formulae [28].

HOMA-IR = [(Fasting serum insulin in U/l x Fasting blood glucose in mmol/l)/22.5]

HOMA-β = (Fasting serum insulin in U/l x 20/Fasting blood glucose in mmol/l– 3.5)

MCP 1, ICAM 1 and VEGF levels were estimated by the ELISA using commercially available kits (R&D Systems, Inc. USA) and following manufacture’s protocol.

### Biochemical parameters of myocardial tissue

The hearts were excised, cleaned immediately with phosphate buffer saline (ice cold; pH 7.4). Cardiomyocytes were isolated from the immediately decapitated hearts of the experimental rats following the method described by Nair and Nair [30] with little modification [31]. Intracellular ROS production was performed in accordance to the method of LeBel and Bondy [32] employing 2,7-dichlorofluorescein diacetate (DCF) as a probe. The method has been slightly modified as mentioned by Kim et al. [33]. The DCF development was evaluated at the excitation and the emission wavelengths of 488 and 510 nm, respectively in a fluorescence spectrometer (HITACHI, Model No. F4500, Japan). The hearts were homogenized in 0.1 M Tris-HCl-0.001 M EDTA buffer (pH 7.4) and centrifuged (@ 12,000 g; 30 min; 4°C). The supernatants were collected for the biochemical assays. The extent of lipid peroxidation (TBARS level) was estimated following the method of Ohkawa and co-workers [34]. The carbonylation of proteins was measured as per the method described by Uchida & Stadtman [35]. Co-enzymes Q_9_ and Q_10_ were appraised employing HPLC as per standard protocol [36]. The level of reduced glutathione (GSH) was assayed by the method described by Hissin & Hilf [37]. The levels of endogenous redox enzymes viz. catalase (CAT), superoxide dismutase (SOD), glutathione peroxidase (GPx), glutathione-S-transferase (GST) and glutathione-6-phosphate dehydrogenase (G6PD) were assessed as the per standard methods [38]. The degree of DNA fragmentation in the selected tissues was measured by the diphenylamine reaction as described by Lin et al. [39]. DNA oxidation was assessed by HPLC and was denoted as the ratio of 8-OHdG to 2-dG [40]. NAD content was assayed as described by Matsumura and Miyachi [41]. Intracellular ATP concentration was estimated using the commercially available assay kit (Abcam, MA, USA).

### Immunoblotting

The protein samples for specific cellular components (whole cell, cytosolic and nuclear fractions) were separated following standard sequential fractionation process as described by Baghirova et al. [42]. Sample proteins (20 μg) isolated from the cardiac tissues of the experimental animals of different groups were subjected to SDS-PAGE (12%) for the separation of proteins and transferred into nitrocellulose membrane following standard transfer protocol [43]. These membranes were blocked by blocking buffer (containing 5% non-fat dry milk; 1 h; room temperature) and subsequently incubated with primary antibodies anti-PKC β (1:500), anti-PKC ε (1:500), anti-PKC δ (1:500), anti-NF-κB (1:2000), anti-PARP (1:2000) and anti-IκBα (1:2000) at 4°C overnight followed by washing with tris-buffered saline (TBST; containing 0.1% tween 20). The membranes were then subjected to suitable HRP-conjugated secondary antibody (1: 3000) at room temperature (1 h). The blots were finally recognized by 3, 3′-diaminobenzidine tetrahydrochloride (Banglore Genei, India). The membranes were then exposed to mild stripping in stripping buffer containing 1% SDS (pH 2.0) and glycine (25 mM) followed by application of anti-β actin (1:6000) primary antibody (@ 4°C) overnight [44]. The membranes were then rinsed with TBST followed by secondary antibody treatment as mentioned before.

### Histological assessment

Hearts from the animals (normal and experimental) were immediately fixed in formalin (10% buffered) after sacrifice and were processed for paraffin sectioning. Sections (thickness ~ 5 μm) were stained (eosin & hematoxylin) to assess under light microscope [45]. For scanning electron microscopy (SEM), isolated animal tissues were processed for the complete removal of blood. Then, the tissues were subjected to stepwise dehydration process following tissue perfusion and fixation [46,47]. Completely dried heart tissues were embedded in araldite. After hardening, resin blocks were subjected to ultra-microtome cutter for ultra-thin sectioning. The sections were observed under analytical SEM (ZEISS EVO 60 scanning electron microscope, Germany) machine with Oxford EDS detector, Germany.

### Statistical analysis

The experimental data were interpreted by one-way ANOVA and expressed as mean ± SD followed by Dunnett’s t-test using computerized GraphPadInStat (version 3.05), GraphPad software, USA. The significance was considered when p < 0.05.

## Results

### Phytochemical analysis

**Fig 2:**
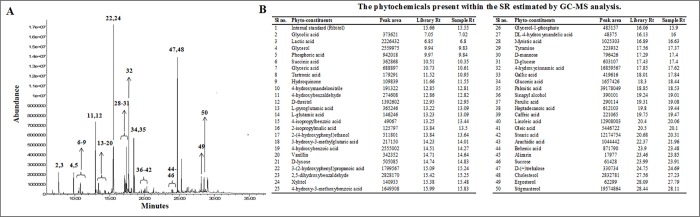
GCMS chromatographic analysis of SR. Panel A. GCMS chromatogram of SR. Panel B. List of identified phytochemicals present within SR. The peaks in Fig 2A have been numbered as per their respective sl no. in Fig 2B.

GC-MS analysis revealed presence of different compounds mainly phenolic compounds, sugar alcohols, sterols, amino acids and saturated fatty acids. The chromatogram and the list of identified compounds have been depicted in Fig 2. Amongst the identified compounds, ferulic acid, caffeic acid, heptadecanoic acid, sinapyl alcohol, gallic acid, 4-hydroxycinnamic acid, 4-hydroxy-3-methoxybenzoic acid, protocatechuic acid, oleic acid, vanillin, hydroquinone, 4-hydroxybenzaldehyde, ergosterol and stigmasterol are important to the context of this study. The importance of the aforementioned compounds has been discussed in the subsequent section of this manuscript.

### Effect on OGTT

**Fig 3:**
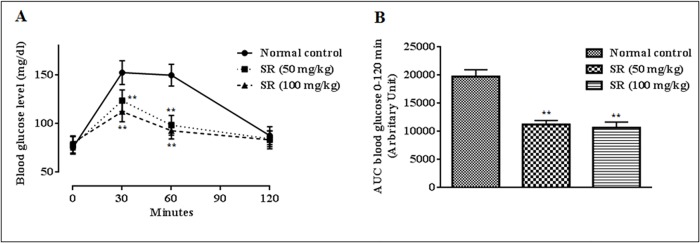
**Effect of SR on oral glucose tolerance test (A); the areas under the curve (AUC) were calculated using the trapezoid method (B).** Data were expressed as mean ± SD (n = 6); *p < 0.05 compared with control group; **p < 0.01 compared with control group.

In order to find out the effect of SR on systemic glucose homeostasis, OGTT has been executed (Fig 3A). The OGTT revealed that, the administration of SR (50, 100 mg/kg) significantly reduced (p < 0.01) blood glucose concentrations between 30–60 min after glucose (1.5 mg/kg) treatment as compared with normal control group. SR also exhibited a significant persuade on total hypoglycaemic response revealed by the significant lessening of AUC as compared with the normal control animals (Fig 3B).

### Effect on fasting blood glucose level

T2D control rats exhibited a significantly raised (p < 0.01) fasting blood glucose level (170 ± 30 mg/dl) before the initiation (Day 0) of the therapeutic regime (Table 2). The principle therapeutic strategy for DM is to maintain the blood glucose level near to normal status. SR (50 and 100 mg/kg) treatment could significantly (p < 0.05–0.01) alleviate fasting blood glucose level, which was observed in the fasting blood glucose levels from day 3 onward. Significant reduction of fasting blood glucose levels were observed on day 3 following SR treatment with the values of ~ 16.1% (p < 0.05) and ~ 17.9% (p < 0.01) for the doses of 50 and 100 mg/kg, respectively (compared to that of fasting blood glucose level in day 0). The experimental observation revealed gradual decrease (p < 0.01) in fasting blood glucose levels following SR treatment in either of the selected doses. However, maximum therapeutic efficacy was observed on 28^th^ day of treatment with a decrease of ~ 25.7% (p < 0.01) and ~ 37.4% (p < 0.01) for the doses of 50 and 100 mg/kg body weight, respectively. The standard drug glibenclamide (1 mg/kg) showed maximum decrease of ~ 48.1% (p < 0.01) on day 28 (Table 2).

**Table 2:** Effect of SR on fasting blood glucose level of T2D rats.

Groups	Fasting blood glucose level (mg/dl) in days						
	0	1	3	7	14	21	28
Group I	76.01 ± 5.94	74.28 ± 5.53	77.04 ± 4.72	76.39 ± 6.11	74.94 ± 3.27	76.50 ± 7.24	75.22 ± 4.56
Group II	171.94 ± 17.71^#^	173.16 ± 13.58^#^	176.76± 13.03^#^	184.31± 19.84^#^	186.12 ± 18.79^#^	193.23± 18.62^#^	191.88 ± 16.67^#^
Group III	173.15 ± 13.06^#^	166.27 ± 14.59^#^	145.22 ± 15.57*	131.04 ± 16.64**	133.04 ± 15.09**	130.15 ± 16.63**	128.67 ± 13.21**
Group IV	173.03 ± 15.90^#^	159.28 ± 11.95^#^	142.11 ± 14.88**	125.11± 18.09**	118.07 ± 14.01**	112.69 ± 12.71**	108.39 ± 14.55**
Group V	172.38 ± 13.22^#^	166.07 ± 16.02^#^	139.08 ± 17.35**	114.27 ± 14.18**	107.61 ± 8.69**	96.33 ± 11.07**	89.44 ± 10.33**

Data were expressed as mean ± SD (n = 6).

^#^p< 0.01 compared with Group I

*p< 0.05 compared with Group II

**p< 0.01 compared with Group II.

Group I: Normal control; Group II: T2D control, Group III: T2D rats treated with SR (50 mg/kg, p.o.); Group IV: T2D rats treated with SR (100 mg/kg, p.o.); Group V: T2D rats treated with glibenclamide (1 mg/kg, p.o.).

### Effects on serum biochemical parameters

The effects of SR on serum biochemical parameters have been presented in Table 3. Significantly increased levels of total cholesterol (p < 0.01) and triglycerides (p < 0.01) in the T2D rats would corroborate the relationship between hyperlipidemia and hyperglycemia. T2D rats exhibited significantly (p < 0.01) low level of serum HDL cholesterol with concomitant increment (p < 0.01) of LDL cholesterol level. However, SR (100 mg/kg) treatment could significantly reinstate the serum lipid (p < 0.05–0.01) levels in T2D rats to near normal status. In this study, T2D rats displayed a significantly (p < 0.01) high level of glycosylated-haemoglobin. An elevated blood glucose concentration in T2D rats is accountable to the up-regulation of glycosylation of proteins. However, SR (100 mg/kg) treatment significantly (p < 0.05) attenuated the glycosylation of haemoglobin to near normal status, which may be due to hypoglycemic effect of SR. The significantly (p < 0.01) raised serum levels of membrane bound enzymes, LDH and CK, revealed the cellular injury due to disintegration of sarcoplasmic membrane. SR (50 and 100 mg/kg) could significantly reduce T2D mediated cellular damage resulting significantly (p < 0.05) reduced levels of CK and LDH in sera. In this study, C-reactive protein level was significantly (p < 0.01) elevated in the sera of T2D animals. An increased level of C-reactive protein stipulated the occurrence of inflammatory disturbances, however, treatment with SR (50 and 100 mg/kg) could significantly (p < 0.01) decrease the C-reactive protein levels in T2D rats. Serum levels of troponins I and T are considered to be the specific markers for myocardial cell injury. The significant increases in the levels of serum troponins I (p < 0.05) and T (p < 0.01) were observed in T2D rats. SR (100 mg/kg) treatment could significantly attenuate the serum troponins I (p < 0.05) and T (p < 0.01) levels in T2D rats.

**Table 3:** Effect of SR on serum lipid profile, glycosylated haemoglobin, membrane bound enzymes, C-reactive proteins and troponin levels of T2D rats.

Parameters	Group I	Group II	Group III	Group IV	Group V
**Total cholesterol (mg/dl)**	92.33± 6.54	156.48 ± 13.21^#^	118.67 ± 9.87*	112.89 ± 6.21**	105.50 ± 8.62**
**HDL cholesterol (mg/dl)**	31.21 ± 3.12	17.67 ± 2.11^#^	25.43 ± 2.85	27.86 ± 2.09*	27.98 ± 2.92*
**Triglycerides (mg/dl)**	116.75 ± 14.56	202.37 ± 19.22^#^	156.88 ± 17.65	138.76 ± 14.32*	133.56 ± 15.67*
**LDL cholesterol (mg/dl)**	37.78 ± 3.45	174.22 ± 9.67^#^	61.47 ± 5.11**	57.28 ± 4.98**	50.81 ± 5.23**
**Glycosylated haemoglobin(mg/g haemoglobin)**	0.32± 0.11	0.63 ± 0.25^#^	0.41 ± 0.16	0.38 ± 0.07*	0.34 ± 0.09**
**Lactate dehydrogenase (U/l)**	187.08± 12.33	285.07 ± 21.15^#^	218.56 ± 17.92*	215.34 ± 18.50*	202.58 ± 20.80*
**Creatine kinase (IU/mg of protein)**	9.42 ± 1.45	19.05 ± 2.04^#^	13.24 ± 1.01*	12.67 ± 1.31*	12.33 ± 1.29**
**C-reactive protein (mg/dl)**	1.14 ± 0.48	3.01 ± 0.72^#^	1.67 ± 0.35**	1.41 ± 0.48**	1.32 ± 0.29**
**Troponin I (ng/ml)**	0.045 ± 0.014	0.087± 0.02^$^	0.06 ± 0.017	0.048 ± 0.028*	0.048 ± 0.033*
**Troponin T (ng/ml)**	0.012 ± 0.002	0.027 ± 0.003^#^	0.02 ± 0.007*	0.017 ± 0.0008**	0.015 ± 0.003**

Data were expressed as mean ± SD (n = 6).

^$^p< 0.05 compared with Group I

^#^p< 0.01 compared with Group I

*p< 0.05 compared with Group II

**p< 0.01 compared with Group II.

Group I: Normal control; Group II: T2D control, Group III: T2D rats treated with SR (50 mg/kg, p.o.); Group IV: T2D rats treated with SR (100 mg/kg, p.o.); Group V: T2D rats treated with glibenclamide (1 mg/kg, p.o.).

**Fig 4:**
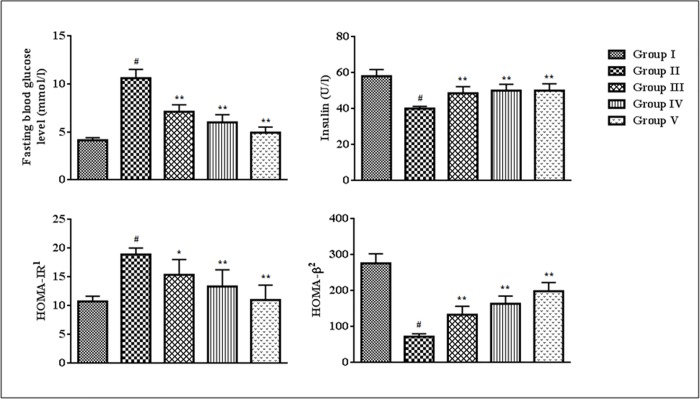
Effect of SR on blood glucose^a^, serum insulin, HOMA-IR and HOMA-β. Data were expressed as mean ± SD (n = 6). ^#^p < 0.01 compared with Group I; *p < 0.05 compared with Group II; **p < 0.01 compared with Group II. Group I: Normal control; Group II: T2D control, Group III: T2D rats treated with SR (50 mg/kg, orally); Group IV: T2D rats treated with SR (100 mg/kg, orally); Group V: T2D rats treated with glibenclamide (1 mg/kg, orally). ^1^HOMA-IR = [(Fasting serum insulin in U/l x Fasting blood glucose in mmol/l)/22.5] ^2^HOMA-β = (Fasting serum insulin in U/l x 20/Fasting blood glucose in mmol/l– 3.5) ^a^ The blood glucose levels used in these assessments were estimated 24 h before sacrificing the animals. Considering the overall duration of the experiment, it has been postulated that the glucose concentration will not vary significantly within 24 h after 28 days of post-treatment.

In this study, T2D rats exhibited significantly lower (p < 0.01) level of serum insulin and HOMA-β score as compared to normal rats (Fig 4). However, a significantly high (p < 0.01) HOMA-IR score was observed in T2D rats (Fig 4). 28-day treatment of SR (50 and 100 mg/kg) could significantly reversed serum insulin level (p < 0.01), HOMA-IR (p < 0.05–0.01) and HOMA-β (p < 0.01) scores near to normalcy (Fig 4).

### Effects on vascular inflammatory markers

**Fig 5:**
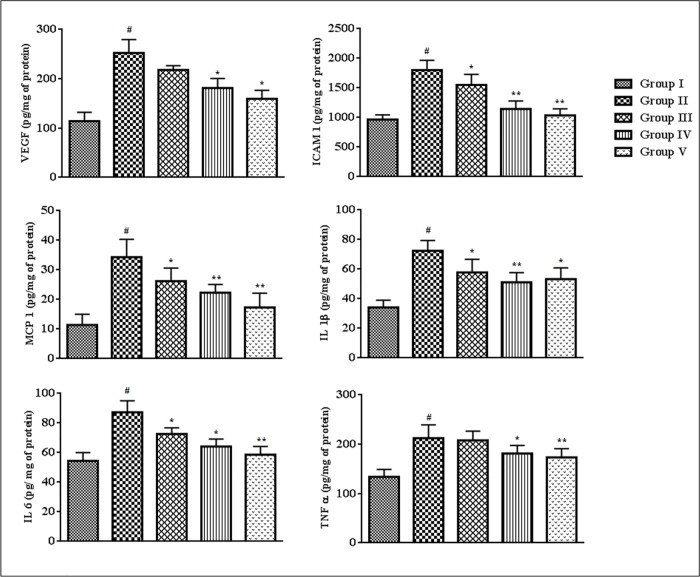
Effect of SR on inflammatory markers viz. VEGF, ICAM 1, MCP 1, IL 1β, IL 6 and TNF α in the sera of T2D rats. Data were expressed as mean ± SD (n = 6). ^#^p < 0.01 compared with Group I; *p < 0.05 compared with Group II; **p < 0.01 compared with Group II. Group I: Normal control; Group II: T2D control, Group III: T2D rats treated with SR (50 mg/kg, orally); Group IV: T2D rats treated with SR (100 mg/kg, orally); Group V: T2D rats treated with glibenclamide (1 mg/kg, orally).

The effects of SR on the vascular inflammatory markers have been estimated in this study (Fig 5). VEGF, ICAM 1, MCP 1, IL 1β, IL 6 and TNF α levels in the sera of T2D rats were significantly (p < 0.01) up-regulated, which revealed the occurrence of vascular inflammation in T2DM. Treatment with SR (50 and 100 mg/kg) could significantly (p < 0.05–0.01) attenuate the expressions of the ICAM 1, MCP 1, IL 1β and IL 6 in the sera of T2D rats, while, VEGF and TNF α levels were significantly (p < 0.05) attenuated at the dose of 100 mg/kg of SR.

### Effects on body weight

In this study, total body weight of experimental rats under different groups was evaluated (Table 4). A significant (p < 0.01) increase of total body weight was observed in T2D rats. SR (100 mg/kg) treatment significantly (p < 0.05) reduced the weight gain of T2D rats. The effect of SR (100 mg/kg) was comparable to that of glibenclamide (1 mg/kg) treated animals.

**Table 4:** Effect of SR on body weight of T2D rats.

Groups	Body weight (g)
Group I	146.54 ± 12.34
Group II	192.50 ± 17.33^#^
Group III	178.65 ± 16.43
Group IV	170.33 ± 12.67*
Group V	169.65 ± 11.25*

Data were expressed as mean ± SD (n = 6).

^#^p< 0.01 compared with Group I

*p< 0.05 compared with Group II.

Group I: Normal control; Group II: T2D control, Group III: T2D rats treated with SR (50 mg/kg, p.o.); Group IV: T2D rats treated with SR (100 mg/kg, p.o.); Group V: T2D rats treated with glibenclamide (1 mg/kg, p.o.).

### Effects on ROS production, protein carbonylation, lipid peroxidation and co-enzymes Q levels in the cardiac tissues

**Fig 6:**
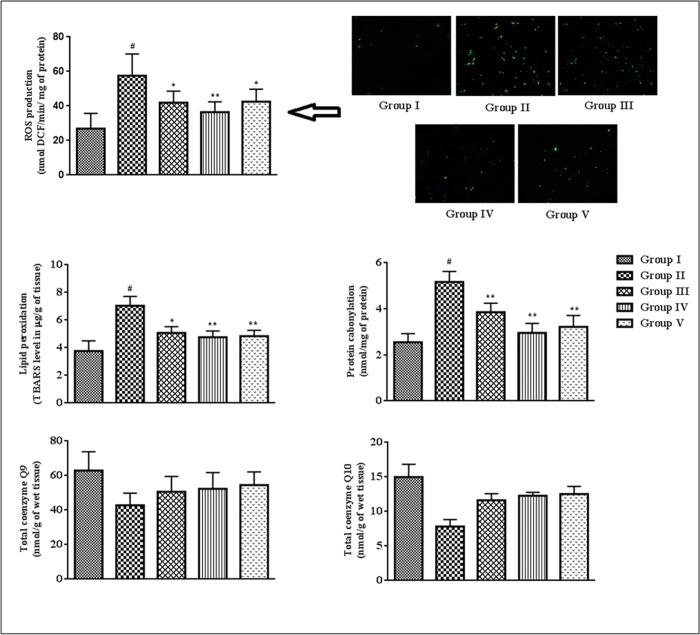
Effect of SR on ROS production, lipid peroxidation, protein carbonylation, coenzymes Q levels in the myocardial tissues of T2D rats. Data were expressed as mean ± SD (n = 6). ^$^p < 0.05 compared with Group I; ^#^p < 0.01 compared with Group I; *p < 0.05 compared with group II; **p < 0.01 compared with Group II. Group I: Normal control; Group II: T2D control, Group III: T2D rats treated with SR (50 mg/kg, orally); Group IV: T2D rats treated with SR (100 mg/kg, orally); Group V: T2D rats treated with glibenclamide (1 mg/kg, orally).

In this study, the degree of lipid peroxidation, co-enzymes Q levels, ROS production and protein-carbonylation in the cardiac tissues were estimated (Fig 6). T2D rats revealed significantly high (p < 0.01) levels of intercellular ROS in the cardiac tissue. SR (50, 100 mg/kg) treatment significantly (p < 0.05–0.01) arrested hyperglycemia mediated ROS generation in the myocardial tissues. The levels of TBARS (a by-product of lipid peroxidation) and carbonylated proteins were significantly (p < 0.01) augmented in the myocardial tissues of T2D rats. SR (50 and 100 mg/kg) treatment, however, could significantly attenuate the extents of protein carbonylation(p < 0.01) and lipid peroxidation (p < 0.05–0.01). T2D rats displayed significantly (p < 0.05–0.01) decreased levels of co-enzyme Q9and Q10in the cardiac tissue (Fig 5). Treatment with SR (100 mg/kg) significantly (p < 0.05–0.01) restored these alterations of coenzymes Q in the heart of T2D rats.

### Effects on endogenous redox markers

**Fig 7:**
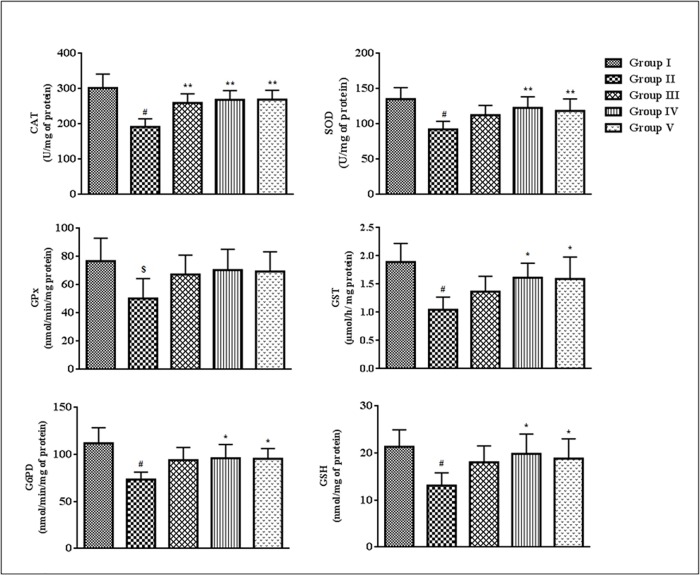
Effect of SR on endogenous antioxidant enzymes (SOD, CAT, GPx, GST, G6PD) and GSH levels in the myocardial tissues of T2D rats. Data were expressed as mean ± SD (n = 6). ^$^p < 0.05 compared with Group I; ^#^p < 0.01 compared with Group I; *p < 0.05 compared with Group II; **p < 0.01 compared with Group II. Group I: Normal control; Group II: T2D control, Group III: T2D rats treated with SR (50 mg/kg, orally); Group IV: T2D rats treated with SR (100 mg/kg, orally); Group V: T2D rats treated with glibenclamide (1 mg/kg, orally).

The effects on endogenous antioxidant enzymes and GSH levels measured in homogenates of the cardiac tissues have been depicted in Fig 7.The levels of CAT, SOD, GPx, GST, G6PD and GSH were significantly (p < 0.05–0.01) decreased in the myocardial tissues of T2D rats as compared with normal animals. Treatment with SR (100 mg/kg) significantly (p < 0.05–0.01) improved CAT, SOD, GST, G6PD and GSH levels of T2D rats, while, no substantial improvement was noticed in GPx level.

### Effects on ATP level, NAD level, DNA fragmentation and DNA oxidation

**Fig 8:**
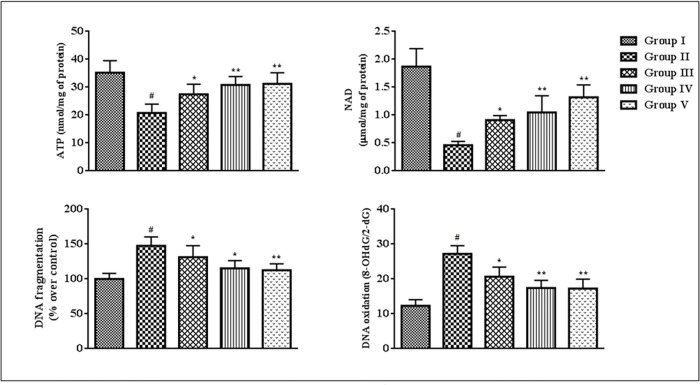
Effect of SR on ATP level, NAD level, DNA fragmentation and DNA oxidation in the myocardial tissues of T2D rats. Data were expressed as mean ± SD (n = 6). ^#^p < 0.01 compared with Group I; *p < 0.05 compared with Group II; **p < 0.01 compared with Group II. Group I: Normal control; Group II: T2D control, Group III: T2D rats treated with SR (50 mg/kg, orally); Group IV: T2D rats treated with SR (100 mg/kg, orally); Group V: T2D rats treated with glibenclamide (1 mg/kg, orally).

The cellular ATP and NAD concentrations give the primary idea about the cellular pathological incidences. In this study, intracellular ATP and NAD levels were significantly (p < 0.01) reduced in the homogenates of the cardiac tissues of T2D rats when compared to that of normal rats (Fig 8). However, treatment with SR (50 and 100 mg/kg) could significantly (p < 0.05–0.01) enhance intracellular ATP and NAD contents in the myocardial tissues of T2D rats. The DNA damage and PARP activation play an essential role in diabetic pathophysiology. In current study, the extents of DNA fragmentation and the oxidation of cellular DNA were significantly increased in the myocardial tissues of T2D rats (Fig 8). However, SR (50 and 100 mg/kg) treatment significantly (p < 0.05–0.01) attenuated the fragmentation and oxidation of DNA in the cardiac tissues of T2D rats as compared with diabetic control animals. The DNA-protective effect would substantiate the overall cyto-protective potential of SR.

### Effects on signal proteins

**Fig 9:**
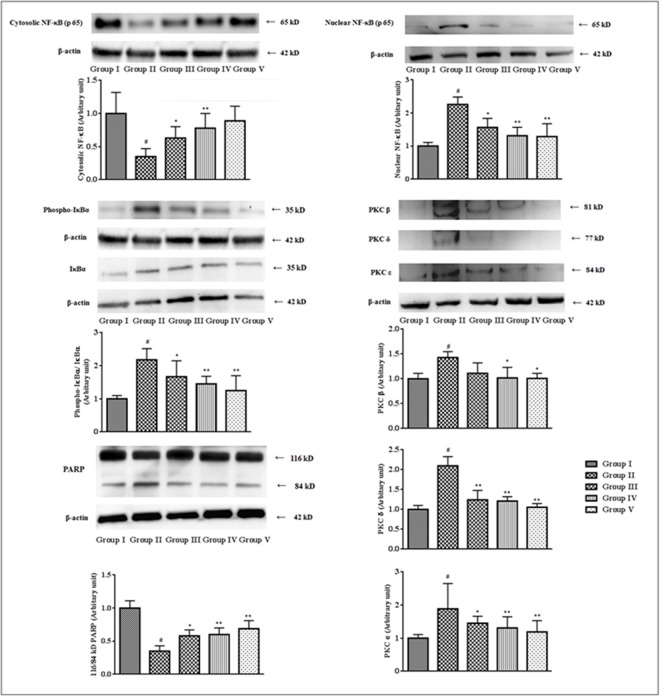
Effect of SR on the expressions of NF-κB, IκBα, PKC isoforms, PARP in the myocardial tissues of T2D rats. The relative band strengths were determined and the intensities of normal control (Group I) bands were given the random value of 1. β actin was used as a loading protein. Data were expressed as mean ± SD (n = 6). ^$^p < 0.05 compared with Group I;^#^p < 0.01 compared with Group I; *p < 0.05 compared with Group II; **p < 0.01 compared with Group II. Group I: Normal control; Group II: T2D control, Group III: T2D rats treated with SR (50 mg/kg, orally); Group IV: T2D rats treated with SR (100 mg/kg, orally); Group V: T2D rats treated with glibenclamide (1 mg/kg, orally).

Activations of various PKC isoforms contribute in many vascular and cellular pathophysiologies. PKCs also participate in the activation of NF-κB under redox challenged environment. In this study, significant (p < 0.01) up-regulations of PKC-β, PKC-δ and PKC-ε were observed in the myocardial (Fig 9) tissues of T2D rats. However, the treatment with SR (100 mg/kg) could significantly (p < 0.05–0.01) attenuate the expression of aforementioned PKC isoforms in T2D rats. Intracellular oxidative challenge activates PARP cleavage which actively participates in the NF-κB activation and DNA damage. In this study, PARP cleavage (p < 0.01) from its full length form (116 kDa) to the cleaved form (84 kDa) was observed in the myocardial tissues of T2D rats (Fig 8). However, extract treatment significantly (p < 0.01) inhibited PARP cleavage. NF-κB, a redox sensitive protein, participates in the instruction of various inflammatory responses. In this study, immunoblottings revealed significant (p < 0.01) up-regulation of nuclear NF-κB (p 65) with concomitant down-regulation (p < 0.01) of cytosolic NF-κB (p 65) in the cardiac tissues of T2D rats (Fig 9). The observation suggested that the translocation of the NF-κB (p 65) to the nucleus, which is crucial for the activation of NF-κB to participate in T2D pathogenesis. The western blot analysis of IκBα revealed IκBα phosphorylation was significantly (p < 0.01) up-regulated in the cytosol of myocardial tissues of T2D rats, which may be correlated to the activation of NF-κB mediated pathogenesis.

### Histological and ultra-structural assessments

**Fig 10:**
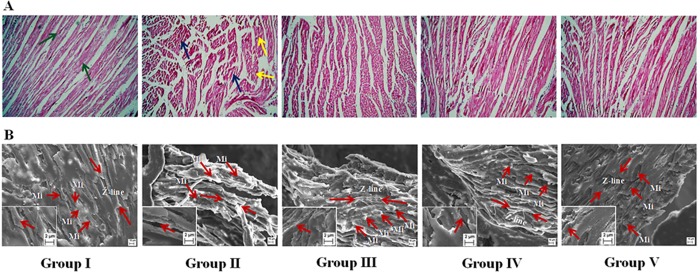
**Histological (Panel A) and ultrastructural (Panel B) assessments of heart of T2D rats of different groups.** Group II exhibited degeneration of interstitial tissues (blue arrows) and change in normal radiating pattern (yellow arrows) in the section of heart, while, Group I exhibited general radiating pattern of heart section. SEM showed ventricular portion of araldite sectioned rat myocardial tissues. Myocardial tissue of normal rats (Group I) exhibited normal myocardial fine structure, with myofibrils comprising regular and continuous sarcomeres which demarcated by Z-lines (Red arrow heads), which were in register with adjacent myofibrils and the rows of moderately electron dense mitochondria (Mi) intervene between myofibrils, while, Group II showed randomly distributed mitochondria (Mi) between poorly organized myofibrils in an electron-lucent sarcoplasm. Group III, IV and V indicated significant improvement in myofibrillar arrangement in heart tissues comparable to that of Group I. Group I: Normal control; Group II: T2D control; Group III: T2D rats treated with SR (50 mg/kg, orally); Group IV: T2D rats treated with SR (100 mg/kg, orally); Group V: T2D rats treated with glibenclamide (1 mg/kg, orally).

The histological heart sections (x 100) of T2D rats revealed the irregular radiating pattern with injured interstitial tissues (Fig 10A). The SEM analyses of hearts of the rats under different groups have been depicted in Fig 10B. Ultrastructural changes of striated muscle of the heart of T2D rats revealed the myofibrillar disorganization. However, treatment with SR could decrease the T2DM mediated histological and ultra-structural aberrations and reinstate the tissue morphology near to normalcy.

The observed effects of SR (50 and 100 mg/kg) were compared with standard drug, glibenclamide (1 mg/kg). The hypoglycemic and hypolipidemic effects of SR (100 mg/kg) were comparable to that of glibenclamide (1 mg/kg). However, SR (100 mg/kg) often exhibited better responses specifically in controlling radox imbalance in T2D rats than the standard drug. Finally, an obese control group was also included in this study to perceive the effect of high fat diets to the experimental rats (S1 Table, S1 Fig). The obese control rats were compared with T2D control and normal control groups. The obese control rats exhibited significantly (p < 0.01) high lipid content in the sera when compared with normal rats. However, the values were also significantly (p < 0.01) differing from T2D rats. The serum insulin level was found to slightly higher (statistically insignificant) in obese control rats when compare with normal rats, however, serum insulin level remained significantly (p < 0.01) high when compared with T2D rats. Obese control rats also exhibited a significant (p < 0.05) increase in fasting blood glucose level when compared with normal control rats, which would have been correlated to the insulin resistance. However, the levels of membrane bound enzymes, glycosylated haemoglobin and C-reactive proteins in the sera remained near normal status. Observing the normalcy in the level of C-reactive proteins in the sera, we did not measure the levels of pro-inflammatory mediators. We also compared the effects of high fat diets in the myocardial tissues (S1 Fig). The experimental data revealed that slight (statistically insignificant) disturbances in the intracellular redox status in the myocardial tissues of obese control rats when compared with normal control rats. However, the tissue parameters were significantly (p < 0.05–0.01) varied in obese control rats when compared with T2D rats.

## Discussion

OGTT gives an idea about glucose-insulin homeostasis under different physiological/clinical states. In this study, OGTT was performed prior to the induction of diabetes. OGTT data revealed that the animals developed hyperglycemia to that experimental rats caused by direct glucose feeding, while, SR treatment could reinstate this effect. It would be possible that, SR might cause an improvement of glucose homeostasis through peripheral glucose uptake [48]. Earlier reports revealed that, the phenolic compounds could attenuate intestinal glucose absorption [49, 50]. Therefore, presence of phenolic substances within SR might also attribute for the overall OGTT observation. The observed OGTT data could predict the probable hypoglycemic effect of SR. Therefore, SR (50 and 100 mg/kg) was subjected to antidiabetic assay employing established T2D model in experimental rats.

High fat diets are the major cause of obesity with simultaneously insulin resistance in the western countries [51]. Streptozotocin has a preferential toxicity toward pancreatic β-cells of islet of Langerhans. Despite the presented literature revealed that β-cells have the ability to regenerate, however, controversies are still existing [52,53]. The partial destruction of β-cells by the small dose of streptozotocin to high fat fed rats has been claimed to induce T2D by lowering insulin secretion coupled with insulin resistance [23,54]. The significantly lower level of serum insulin in T2D control rats indicted the partial destruction of pancreatic β-cells. Besides, significantly low HOMA-β value and significantly high HOMA-IR value in T2D control rats established the induction of insulin resistance [28]. Therefore, high fat diets + low single dose of streptozotocin model has been claimed to be an optimum experimental model for T2D simulating the human T2DM [23], which has been employed in this study to evaluate protective effect of SR.

In this study, the animals were divided into five groups. Group I and II represented normal and T2D animals, respectively. The T2D mediated pathological changes were statistically compared normal animals. Groups III and IV were kept as test groups to observe the protective role of SR. The studied parameters of test groups were statistically compared with respect to T2D control group. Group V represented positive control animals to compare the overall protective effect of SR with respect to commercially available oral hypoglycemic agent, glibenclamide.

Reduction of the blood glucose level is the principle approach of diabetic therapy. Inclusion of low dose of streptozotocin caused incomplete destruction of β-cell population in islet of Langerhans. In this study, significant reduction of serum insulin level was observed. Insulin is known to activate lipoprotein lipase which catalyses the hydrolytic breakdown of lipids during normal physiological status [3]. Therefore, lower insulin level coupled with insulin resistance during diabetic condition causes hyperlipidemia. In this study, high concentrations of serum lipids were observed in T2D rats. SR treatment could significantly reverse HOMA-β and HOMA-IR scores with concomitant promotion of insulin secretion. SR treatment could significantly attenuate hyperlipidemia, which would be corroborated with the reversal of insulin resistance coupled with elevation of insulin secretion. Persistent hyperglycemia promotes glycosylation of different functional proteins including haemoglobin [3]. In this study, a significant elevation in the level of glycosylated haemoglobin was observed in the sera of T2D rats. Increased CK and LDH contents in the sera are primary indication of cellular damage [55]. These membrane bound enzymes come into the blood during cellular injury. In this study, CK and LDH levels in the sera were significantly raised in T2D rats over control, which revealed the occurrence of hyperglycemia mediated cytotoxicity. SR treatment significantly reduced the levels of CK and LDH in the sera of T2D rats, which indicated the cyto-protective role of test extract during DM.

Increased blood glucose level facilitates generation of ROS which directly participate in the pathological incidences in DM. Cardiovascular injury is a critical reason of morbidity and mortality of the DM patients [4]. Earlier reports revealed that hyperglycemia mediated excessive ROS generation plays predominant role in diabetic cardiomyopathy [3,4]. In this study, a significantly high ROS production was observed in cardiac tissues of T2D rats. An enhanced generation of ROS would result in the increases in lipid peroxidation, protein carbonylation with concomitant depletion of endogenous antioxidant molecules [55,56]. Therefore, it would be concluded that myocardial tissues experienced to redox challenge/oxidative stress during DM. SR treatment could significantly attenuate intracellular ROS levels in the myocardial tissues of T2D rats. SR could produce the effect either by direct scavenging ROS and/or indirectly by inhibiting ROS generation through its hypoglycemic effect. A decrease in the levels of ROS in the myocardial tissues in SR treated T2D rats caused the reduction of peroxidative damages of cellular lipids and carbonylation of proteins. SR also ensured better protection against oxidative stress by up-regulating endogenous antioxidant molecules. In a redox challenged cellular environment, an excessive amount of GSH is utilized and subsequently GSH level is decreased [4]. Later encourage generation of many reactive intermediates which cause DNA damage and cell death. The hyperglycemic rats exhibited a significantly increased level of 8-OHdG/2-dG ratio, an index of DNA oxidation and DNA fragmentation. However, SR could significantly prevent DNA oxidation and fragmentation, which would be due to radical scavenging effect synergized with hypoglycemic effect of test material.

Hyperglycemia mediated oxidative stress could simultaneously activate PKCs by the influx of the polyol pathway [57]. Activation of PKC isoforms contributes in the activation of NF-κB in redox challenged cellular environment. PKCs also largely contribute to the accumulation of matrix proteins like collagen and cause fibrosis [4]. In this study, the expressions of PKC β, δ and ε were significantly up-regulated in the myocardial tissues of T2D rats. However, SR treatment significantly reversed the elevated expressions of PKC isoforms in the myocardial tissues of T2D rats. Intracellular oxidative pressure potentiates PARP cleavage which further promotes the activation of NF-κB [58]. NF-κB is one of the redox sensitive proteins, which participates a crucial role in the inflammation process [3]. Oxidative stress causes degradation of IκBα via phosphorylation with concomitant translocation of NF-κB to the nucleus from cytosol [58]. Translocated NF-κB binds with DNA and regulates the expressions of several molecules like pro-inflammatory cytokines, VEGF, ICAM 1 related to diabetic pathophysiology [4]. In this study, T2D rats exhibited up-regulated expression of NF-κB in nucleus of cardiac tissues following release of inflammatory mediators. However, SR treatment could significantly attenuate the NF-κB mediated inflammatory responses.

GC-MS analysis revealed presence of phenolic compounds, phenolic acids, fatty acids and sterols in SR. The different compounds present within the SR have been reported to display hypoglycemic, anti-inflammatory and antioxidant effects which have been discussed hereunder. Ferulic acid manifests antidiabetic potential by modulating insulin-signaling molecules [59]. Caffeic acid possesses significant antidiabetic activity [60]. Besides, caffeic acid and its derivatives exhibited significant anti-inflammatory effect via antioxidant mechanism [61]. Oleic acid has been reported to counteract with the inhibitory effect of inflammatory cytokines in insulin production [62]. Ergosterol has been reported to possess significant hypoglycemic effect and counteract with diabetic pathophysiology via inhibiting NF-κB mediated inflammatory signals [63]. Stigmasterol is also known to possess hypoglycemic effect [64]. Heptadecanoic acid, a saturated fatty acid, has been reported to reverse pre-diabetes condition [65]. Sinapyl alcohol has been proposed to inhibit LPS stimulated TNF-α production [66]. Gallic acid has been reported to exhibit cardioprotective effect via redox balancing in experimentally induced diabetic rats [67]. 4-hydroxycinnamic acid has been reported to possess hypoglycemic and hypolipidemic effect in diabetic rats [68]. Protocatechuic acid exhibited significant antidiabetic, anti-inflammatory and antioxidant effects [69]. 4-hydroxy-3-methoxybenzoic acid has been reported to possess hypoglycemic effect [70]. Vanillin has been reported to attenuate the expressions of pro-inflammatory cytokines via anti-oxidant mechanism [71]. Hydroquinone and 4-hydroxybenzaldehyde have been reported to exhibit anti-inflammatory effect [72,73]. Besides, a significant number of phenolic acids within SR would attribute significant radical scavenging effect in diabetic pathophysiology. However, the overall effect would be exerted through the synergy between the aforementioned compounds.

## Conclusion

**Fig 11:**
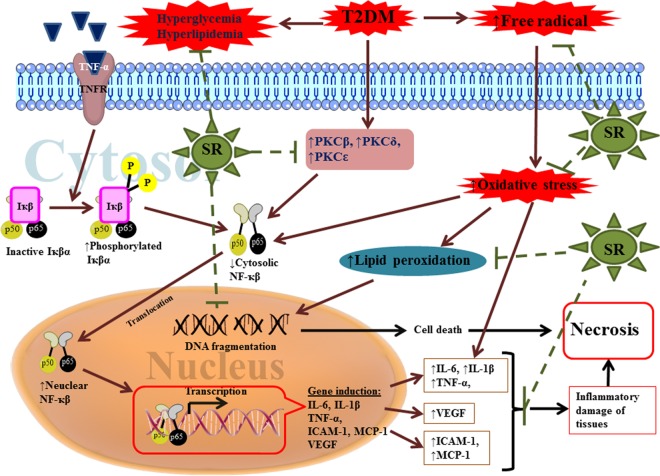
A schematic overview of the hypothesis developed in this study regarding probable protective mechanism of SR against diabetic cardiomyopathy. Green dotted lines represented the restricted pathological events by SR.

DM is associated with hyperglycemia which largely contributes in generation of excess of ROS. Excess of ROS actively initiates and propagates a number of toxicological incidences including diabetic cardiomyopathy. It has been proposed that, ROS activates the expressions of several redox sensitive proteins which contribute in the toxicological process. ROS mediated activation of PKC isoforms, PARP cleavage and NF-κB translocation to the nucleus constitute integrally in the diabetic cardiomyopathy via activation of inflammatory pathway and leading to necrotic cell death. Besides, excess of ROS attack cellular nucleic acids and participate in cell death process. Considering the multiple mechanisms involved in the diabetic cardiomyopathy (Fig 11), a multi-target therapeutic strategy would be fruitful. The experimental outcome of this study clearly suggested that SR could offer overall protective effect through attenuating hyperglycemia, scavenging ROS and arresting inflammation (Fig 11). The observed effect has been correlated with the existing phytochemicals. Therefore, it could be concluded that SR would have potential to be developed as a novel phytotherapeutic agent for T2DM in future.

## Supporting Information

S1 Table Effects on fasting blood glucose and other biochemical parameters in the sera of normal, Type II diabetic and fat fed rats. (DOC)

S1 Fig Effects on fasting blood glucose and other biochemical parameters in the sera of normal, Type II diabetic and fat fed rats. Data were expressed as mean ± SD (n = 6). ^$^p< 0.05 compared with Group I; ^#^p< 0.01 compared with Group I; *p< 0.05 compared with Group II; **p< 0.01 compared with Group II. Group I: Normal control group; Group II: T2D control group, Group VI: Obese control group.
(TIF)
