# Supplementary material for: Sansevieria roxburghiana Schult. & Schult. F. (Family: Asparagaceae) Attenuates Type 2 Diabetes and Its Associated Cardiomyopathy
Source: PLoS One. 2016 Nov 28;11(11):e0167131. doi: 10.1371/journal.pone.0167131 (PMC5125675; doi:10.1371/journal.pone.0167131)
Supplement: S1 Table — (DOC) [file pone.0167131.s001.doc]

**S1 Table.** Effects on fasting blood glucose and other biochemical parameters in the sera of normal, Type II diabetic and fat fed rats.

| **Parameters** | **Group I** | **Group II** | **Group VI** |
| --- | --- | --- | --- |
| **Fasting blood glucose (mg/dl)** | 75.22 ± 4.56 | 191.88 ± 16.67^#^ | 87.75 ± 5.43^$,**^ |
| **Total cholesterol (mg/dl)** | 92.33± 6.54 | 156.48 ± 13.21^#^ | 136.24 ± 10.89^#,**^ |
| **HDL cholesterol (mg/dl)** | 31.21 ± 3.12 | 17.67 ± 2.11^#^ | 24.67 ± 2.33^#,**^ |
| **Triglycerides (mg/dl)** | 116.75 ± 14.56 | 202.37 ± 19.22^#^ | 152.33 ± 14.25^#,**^ |
| **LDL cholesterol (mg/dl)** | 37.78 ± 3.45 | 174.22 ± 9.67^#^ | 81.10 ± 7.63^#,**^ |
| **Insulin (U/l)** | 58.16 ± 3.69 | 40.11 ± 1.24^#^ | 65.22 ± 4.54^**^ |
| **Glycosylated haemoglobin (mg/g haemoglobin)** | 0.32± 0.11 | 0.63 ± 0.25^#^ | 0.36 ± 0.17^**^ |
| **Lactate dehydrogenase (U/l)** | 187.08± 12.33 | 285.07 ± 21.15^#^ | 199.50 ± 16.28^**^ |
| **Creatine kinase (IU/mg of protein)** | 9.42 ± 1.45 | 19.05 ± 2.04^#^ | 11.33 ± 1.34^**^ |
| **C-reactive protein (mg/dl)** | 1.14 ± 0.48 | 3.01 ± 0.72^#^ | 1.21 ± 0.43^**^ |

Data were expressed as mean ± SD (n=6). ^$^p< 0.05 compared with Group I; ^#^p< 0.01 compared with Group I; ^**^p< 0.01 compared with Group II. Group I: Normal control group; Group II: T2D control group, Group VI: Obese control group.
