# Supplementary figures and images for: Sansevieria roxburghiana Schult. & Schult. F. (Family: Asparagaceae) Attenuates Type 2 Diabetes and Its Associated Cardiomyopathy
Source: PLoS One. 2016 Nov 28;11(11):e0167131. doi: 10.1371/journal.pone.0167131 (PMC5125675; doi:10.1371/journal.pone.0167131)

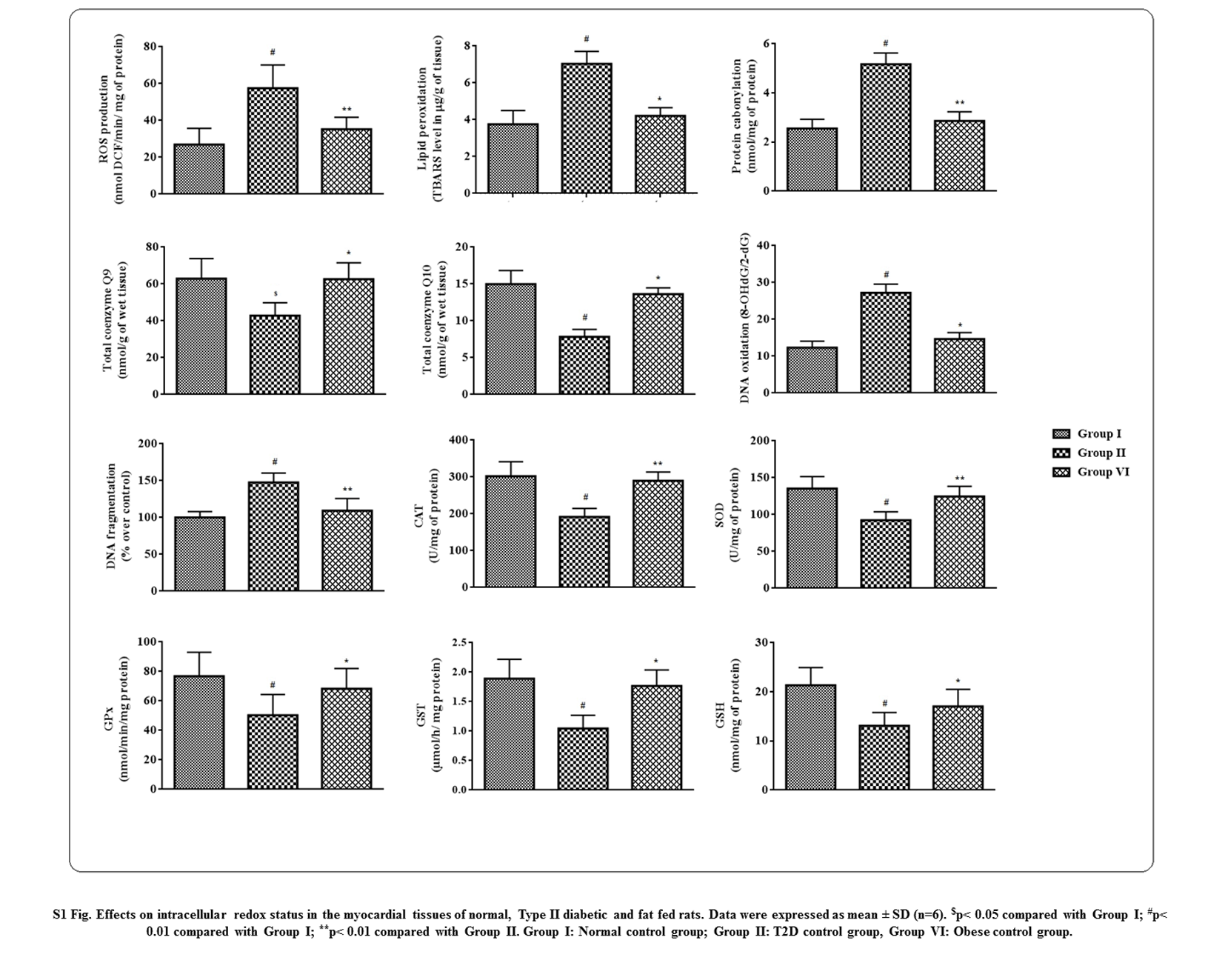

Supplement: S1 Fig — Data were expressed as mean ± SD (n = 6). $p< 0.05 compared with Group I; #p< 0.01 compared with Group I; *p< 0.05 compared with Group II; **p< 0.01 compared with Group II. Group I: Normal control group; Group II: T2D control group, Group VI: Obese control group. (TIF) [file pone.0167131.s002.tif]
